# *Nutrients* 2009–2019: The Present and the Future of Nutrition

**DOI:** 10.3390/nu11010088

**Published:** 2019-01-04

**Authors:** Lluis Serra-Majem, Maria Luz Fernandez

**Affiliations:** 1 Research Institute of Biomedical and Health Sciences, University of Las Palmas de Gran Canaria, 35016 Las Palmas de Gran Canaria, Spain; 2 Consorcio CIBER, M.P. Fisiopatología de la Obesidad y Nutrición (CIBERObn), Instituto de Salud Carlos III (ISCIII), 28029 Madrid, Spain; 3 Department of Nutritional Sciences, University of Connecticut, Storrs, CT 06269, USA

As we move forward and continue to publish the most recent and prominent scientific findings in Nutritional Sciences for the next 10 years, the current editors-in-chief would like to take a look at the history of *Nutrients.* During its first decade, *Nutrients* very promptly became a well-known, heavily cited journal in a short period of time. These accomplishments were possible due to the vision of the founding editors as well as the commitment of the associate editors, editorial board members, and managing editors of the journal.

*Nutrients* was first launched in September 2009 with Professor Peter Howe as its founding editor. It was initially published as a quarterly journal. However, in 2010, *Nutrients* was changed to a monthly publication schedule and the journal was indexed by Scopus. In 2011, *Nutrients* was accepted for indexing by Web of Knowledge and Medline (ISI), and by Science Citation Index Expanded (SCIE) a year later. *Nutrient*’s first impact factor was 0.676. In 2012, the impact factor was 2.072 and 3.148 in 2013 and continued increasing to 3.270 in 2014 and 3.759 in 2015, with a small decrease to 3.550 in 2016, and then climbed to 4.196 in 2017, when it was ranked in the first quartile (18/81 in the category “Nutrition and Dietetics” in SCIE).

In 2015, the first 1000 papers were published, and a year later, 2000 papers were achieved, 3000 in 2017, 4000 in 2018, and we expect to get 5000 early in 2019 to celebrate *Nutrient*’s 10th Anniversary. During this period, more than 130 open supplements have been published, covering the hottest topics in nutrition and metabolism. For next year, these are just some of the topics of the forthcoming supplements, which demonstrate and highlight the broad aspects of nutrition that are published in *Nutrients*: *Nutritional advances in the prevention and management of chronic disease*; *Nutrient targeting of intestinal mucosal wall to modulate metabolism*; *Nutrition and endothelial function*; *Effects of diets and active compounds on nonalcoholic fatty liver disease*; *Vitamin E: uses, benefits, emerging aspects, and RDA*; *Choline as an essential nutrient*; and *Advances in parenteral nutrition*, just to mention a few.

During this decade, *Nutrients* has established alliances with several societies and organizations (Nutrition Society of New Zealand (NSNZ) and Australasian Section, American Oil Chemists Society (AAOCS), Italian Society for Paediatric Nutrition and Gastroenterology (SIGENP), Asia Pacific Nutrigenomics Nutrigenetics Organisation (APNNO), The Nutrition Society of Australia (NSA), International Chair for Advanced Studies on Hydration (CIEAH), and the Spanish Society of Community Nutrition (SENC)) and will continue to approach the leading nutrition and food societies across the globe, including the American Society of Nutrition (ASN). In 2018, *Nutrients* launched “The Young Investigator Award” to encourage young investigators to publish their pioneer work in our journal. Young researchers represent the future of nutrition.

Currently, *Nutrients,* exclusively published in open access, follows strict processing procedures making sure of the quality of published papers, with an extremely short average processing time of 35 days. *Nutrients’* high reputation is based on a solid editor team with an editorial board of devoted and expert associate editors, editorial board members, and reviewers, who guarantee the outstanding level of all accepted papers, and very efficient managing editors. Our most sincere thanks to all of them.

To celebrate its 10th anniversary, *Nutrients* is sponsoring a conference in Barcelona next 27–29 September 2019, where we will have the possibility to exchange the latest news in the nutrition arena. We will also have time to acknowledge the work done in this first decade and to envision the next 10 years with new targets and missions. Thanks to all of you for having chosen *Nutrients* to help us write the present and the future of nutrition expeditiously, transparently, and rigorously.

